# Improved Dynamic Window Approach for Unmanned Surface Vehicles’ Local Path Planning Considering the Impact of Environmental Factors

**DOI:** 10.3390/s22145181

**Published:** 2022-07-11

**Authors:** Zhenyu Wang, Yan Liang, Changwei Gong, Yichang Zhou, Cen Zeng, Songli Zhu

**Affiliations:** 1 School of Ocean Science and Technology, Dalian University of Technology, Panjin 124221, China; 21928009@mail.dlut.edu.cn (Y.L.); gongchangwei@mail.dlut.edu.cn (C.G.); 2 Leicester International Institute, Dalian University of Technology, Panjin 124221, China; tuzi0905@mail.dlut.edu.cn; 3 Division of Human Biology, Fred Hutchinson Cancer Center, Seattle, WA 98109, USA; szhu23@fredhutch.org

**Keywords:** USV, local path planning, DWA, marine environment

## Abstract

The aim of local path planning for unmanned surface vehicles (USVs) is to avoid unknown dynamic or static obstacles. However, current relative studies have not fully considered the impact of ocean environmental factors which significantly increase the control difficulty and collision risk of USVs. Therefore, this work studies two ocean environmental factors, namely, wave and current, given that they both have a significant impact on USVs. Furthermore, we redesign a kinematic model of an USV and the evaluation function of a classical and practical local path planning method based on the dynamic window approach (DWA). As shown by the results of the simulations, the path length was impacted mainly by the intensity of the environmental load and slightly by the direction of the environmental load, but the navigation time was significantly influenced by both. Taking the situation in still water as a benchmark in terms of the intensity and direction of the environmental factors, the maximum change rates of the path length were 8.6% and 0.6%, respectively, but the maximum change rates of the navigating time were 17.9% and 25.6%, separately. In addition, the average calculation time of each cycle was only 0.0418 s, and the longest time did not exceed the simulation time corresponding to a single cycle of 0.1 s. This method has proven to be a good candidate for real-time local path planning of USVs since it systematically considers the impact of waves and currents on the navigation of USVs, and thus ensures that USVs can adjust to the planned path in time and avoid obstacles when navigating in the real ocean environment.

## 1. Introduction

The ocean provides humanity with massive resources, such as petroleum, natural gas, combustible ice, and minerals. With the increasing shortage of land resources, the development and exploration of marine resources are becoming more and more critical for human survival and development. However, due to ocean currents and climate change, there are many unprecedented challenges and problems in the exploration of marine resources. Therefore, unmanned carrier equipment such as unmanned surface vehicles (USVs) have attracted extensive attention, as their unique advantages make marine resource development and exploration safer and more flexible. To realize autonomous navigation, USVs must be optimized in two respects, namely, detecting and positioning ability and path planning. As the former has already been well-developed, the latter has unquestionably become the major constraint on improving the automation level of USVs [1,2,3]. As an essential part of the USV navigation task management system, path planning intensively embodies intelligence level. Given current marine environment and automation requirements, there are three critical issues that need to be considered in the autonomous navigation of USVs in the real marine environment, including safety, the reliability of task completion, and the probability of success. According to the level of understanding of the marine environment, path planning methods can be divided into global path planning and local path planning. Global path planning is realized mainly with the waypoint method, which applies to cases where there are only static obstacles and all environmental information is available in advance, that is, one point is left at each interval and then each adjacent waypoint is connected with a straight line to generate the shortest path without the time and the other parameters. The A* method [4] and D* Lite method [5] are classic global path planning methods, usually with low resolution, and have been applied to autonomous underwater vehicles (AUVs) in early years [6,7,8]. However, AUVs and USVs have differences in functional areas, environmental conditions, running speed, and load capacity. Therefore, it is necessary to develop a specific path planning method for USVs based on the one adopted by AUVs. In the past five years, more and more studies have focused on the global path planning method adopted by USVs in the marine environment [9,10,11], indicating the great potential of this method in the development of USVs.

Unlike global path planning, local path planning is usually conducted via the trajectory-based method. Since the generated path contains more information, such as time parameters, and can better guide the driving of the USV, the local path planning method is suitable for dynamic environments that do not require prior environmental information. High-resolution tracks with moving obstacles can be found even in the navigation process. However, this method can only be executed in real-time rather than in an offline environment, and usually requires many advanced sensors and computing resources. In the local path planning method, the USV also adopts the traditional obstacle avoidance algorithms adopted by mobile robots, such as the artificial potential field method, DWA, and fuzzy control algorithm. Moreover, many studies have introduced more constraint conditions and established more accurate models for USVs. Specifically, Meng et al. improved the safety and global optimality of the DWA considering the nearest dynamic obstacles and global waypoints [12], but they did not investigate the impact of the marine environment. Chen et al. proposed an artificial potential field algorithm based on ant colony optimization for the local and global path planning of unmanned ships in a dynamic environment. The feasibility and effectiveness of the algorithm were verified by a series of field experiments and simulations. However, even though PID and other methods were adopted to control the influence of wind and other environmental factors in these field experiments, there were still significant errors between the actual path and the planned path, with a maximum error of almost 10 m, which fully proves the considerable impact of the marine environment on the path planning algorithm [13].

Some previous studies have indeed taken into account the impact of the marine environment or navigation rules. Specifically, using a rigid body dynamic model of an USV, Mousazadeh et al. carried out a legal obstacle avoidance optimization under wave interference based on searching balls and potential field functions [14]. Song et al. proposed a novel multilayer fast stepping method (MFM) which realized real-time collision avoidance for USVs in an environment with a time-variant ocean current [15]. However, these studies only considered the impact of a single factor one-sidedly—either ocean waves or current—and ignored that the both factors may simultaneously exert an impact on USVs in the real marine environment. Therefore, it is necessary to develop a path planning method that comprehensively considers the impact of both factors.

Significantly, recent studies have begun to comprehensively consider the impact of marine environmental factors on USV path planning. Aiming at the navigation safety and energy consumption of USVs in the complex marine environment, Ding et al. proposed a new energy-saving path planning and path tracking control method based on particle swarm optimization (PSO) [16]. However, in the process of path planning, they neither considered dynamic obstacles nor introduced the impact of the marine environment on the maneuverability of the USV and instead, in the process of PID control, simply conducted dynamic obstacle avoidance and the completion of a planned path, which significantly reduces the practicability of this method. In 2019, Wang et al. presented a multilayer path planner for a USV with global path planning, collision avoidance, and conventional correction, and then simulated it in a complex marine environment with constraints of coastal and sea surfaces [10]. However, the real-time and dynamic obstacle avoidance performance was not ideal because the local path planning method they developed with the B-Spline method could not overcome the common shortcomings of the spline interpolation algorithm. Fang et al. proposed a multi-objective path planning algorithm for USVs based on the S-57 electronic chart and adaptive self-learning particle swarm optimization (AMPSO). According to the requirements of different tasks of USVs, a multi-objective path planning model considering path length, smoothness, energy consumption and marine environment interference was established. Compared with other path planning algorithms, this algorithm has stronger convergence speed and search ability, and takes account of multi-objective optimization. However, this algorithm only focuses on the upper planning level, lacking sufficient combination with the control level, which definitely impedes the completion of path planning [17].

Overall, there are still many problems with the local path planning method, as follows. First, the local path method needs to be adjusted according to the needs of the USV, since there is no general algorithm or theory to regulate the path planning of the whole platform and all fields. Second, most current studies on path planning methods pay more attention to the upper planning level rather than the low control level, which leads to the low rationality of planned paths and difficult navigation. Third and most importantly, the state of the USV during practical navigation in the complex marine environment is entirely different from the ideal state in traditional path planning research. When a USV navigates in a strong ocean current environment, the impact of environmental factors in the path will lead to a large amount of energy consumption, reduce the controllability of the USV and increase the probability of collision with obstacles (moving or stationary) [18].

Therefore, we adopt the classic and practical DWA as the basic algorithm [19,20] and improve the kinematic model and evaluation function in this algorithm, thereby quantitatively introducing the effects of waves and currents into the constraints of path planning and fundamentally improving the enforceability of the planned path. Furthermore, we verify the rationality and effectiveness of the improved method by simulation experiments.

## 2. Method Description

### 2.1. Subsection

To reduce the complexity of USV path planning, some appropriate simplifications should be conducted in practice. Therefore, this study fully considered the actual situation and made the following assumptions:

1. The map (study area) was considered as an independent marine environment. Therefore, we assumed that the impact of environmental factors on USV navigation in the study area was fixed and would not change with time, and meanwhile the marine environment would not be affected by USV navigation;
2. The environment map used in the study was a converted grid map composed of many small squares with a side length of 1. The free passage space and obstacles were represented with white and black separately, and then were defined as 1 and 0, respectively, in the two-dimensional array storing map information. When judging the distance, we simplified USV as a point and correspondingly modeled the square representing the obstacle into a circle with the center of this square as the center and the diameter of greater than or equal to $\sqrt{2}$. In this paper, the value of 1.6 was selected. In this way, the distance between the USV and the obstacle was calculated as the distance from the point to the circle;
3. It was assumed that the position and speed of all obstacles on the map were obtained via the sensors and the offline map of the USV. Considering the speed and controllability of the USV, the distance threshold of obstacles was set as 200 m, so only the unknown obstacles within 200 m from USV were included in the calculation of obstacle avoidance. This method had the risk of causing the algorithm to fall into the optimal local solution, but the risk was not high in the short-distance path planning task. Thus, we saved many computing resources by this method;
4. It was assumed that the given moving obstacle moved in a straight line at a uniform speed, without interaction between the environment and the obstacle;
5. The USV was simplified as a point when calculating the trajectory. In addition, when calculating the collision problem, we beforehand defined the safety distance R that could reflect the size of the USV and the safety rules that were set manually. In this study, the value of R was set as 4 m;
6. Since the result of a previous calculation cycle can be used as the initial condition of the current cycle in the iterative process of the algorithm, we assumed that the USV navigated according to the planned path to ensure the correctness of the subsequent calculation results. We highly support the reasonableness of this assumption given that this study fully considered the actual handling characteristics and the main environmental factors of the USV;
7. There are three main marine environmental factors that can exert an impact on an USV, namely, wind, waves, and ocean currents; these can cause additive or multiplicative interference with the USV [21]. Considering the actual draft, the projected area of the USV in the air was much smaller than that under the water. Therefore, when the wind speed was less than ten m/s, the effect of wind load on the USV was usually not apparent [22], which can be ignored in path planning. Assuming that the fluid pressure only caused the wave load, its interference force and moment on the ship were caused by the fluctuation of the pressure field distribution of the fluid under the water surface [21]. Therefore, the wave load was more important in dynamic path planning than in global path planning. In reality, the ocean current was irregular and multidirectional in space and time, but this study assumed that the ocean current remained unchanged in a given time considering that the calculated sea area was small and the USV transit time was short. Since the ocean current is essentially the movement of water in the ocean, the influence of ocean currents on USVs can be expressed by the superposition of the velocity of the ocean current and the navigation velocity of the USV.

### 2.2. DWA Considering the Marine Environmental Impact

**Figure 1:**
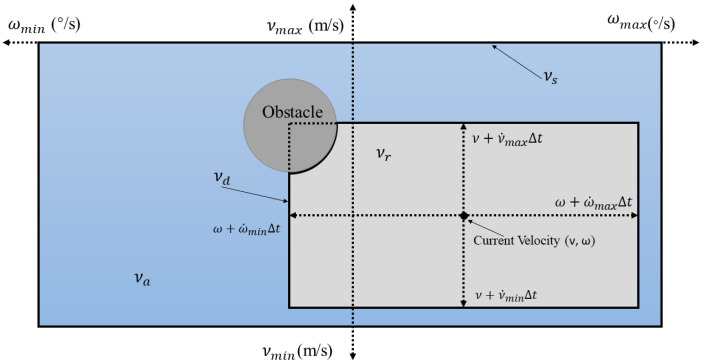
The velocity vector space diagram of the DWA.

In this work, DWA was selected as the basic algorithm given that it can solve the dynamic obstacle avoidance problem. The traditional DWA takes the limitations of velocity and acceleration and aims for a collision free trajectory, thereby forming a dynamic window with sampled velocity. Then, an optimal trajectory was selected from this dynamic window based on the designed evaluation function. The current optimal trajectory was continuously searched by repeating iterations until the target point was reached. Figure 1 is the velocity vector space diagram of the DWA [23,24]. The vertical and horizontal axis represent the linear velocity and the angular velocity, respectively. *V_a_* represents the feasible velocity region to avoid collision. The speed range of the USV was also limited by its own performance, so *V_s_* represents all the speed ranges it can reach and *V_d_* represents the speed ranges it can reach in the next iteration cycle, where *V_d_* is expressed by Equation (1).

(1) $$V_{d}=\left\{\left(v,\omega \right)|v\in \left[\nu +{\dot{v}}_{min}\Delta t,\nu +{\dot{v}}_{max}\Delta t\right],\omega \in \left[\omega +{\dot{\omega }}_{min}\Delta t,\omega +{\dot{\omega }}_{max}\Delta t\right]\;\right\}$$

Therefore, the intersection *V_r_* comprising the three sets of *V_s_*, *V_d_* and *V_a_* represents the optional velocity range of the USV, namely, the final dynamic window.

(2) $$V_{r}=V_{s}\cap V_{a}\cap V_{d}$$

Based on the evaluation function, we selected the trajectories corresponding to the optimal evaluation value from multiple groups of trajectories, and then found the driving angular velocity and linear velocity of this trajectory. Therefore, the DWA can be divided into the kinematic model, speed sampling, and evaluation function. In this study, the impact of the environment was reflected in the establishment of the kinematic model and evaluation function.

#### 2.2.1. Kinematic Model

In the DWA, the optimal angular velocity and linear velocity group are selected depending on the simulation of the USV trajectory; thus, it was necessary to establish the corresponding kinematic model according to the existing USV. It was assumed that under the selected linear velocity and angular velocity the motion trajectory of USV was a circular arc, and that it was a straight line when the value of angular velocity was zero. In this way, we focused on studying the impact of environmental factors on the USV.

- Kinematic model in still water

**Figure 2:**
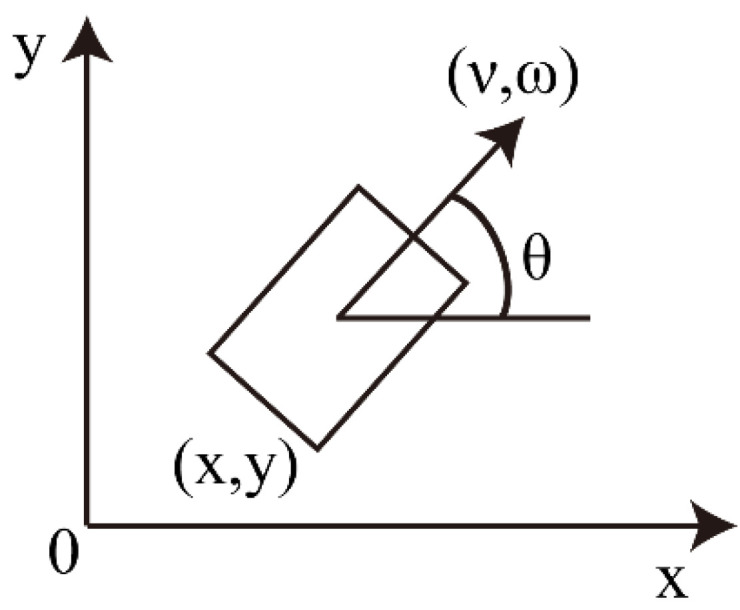
Schematic diagram of the USV kinematic model.

A USV is a typical under-actuated system which only has forward and steering capabilities without lateral driving force. At time *t*, the coordinates of the USV in the world coordinate system are represented by *x*(*t*) and *y*(*t*), and the direction angle (heading angle) of USV is represented by *θ*(*t*). As shown in Figure 2, the group of (*x, y, θ*) represents the kinematic position and pose of the USV.

Meanwhile, at time *t*, the linear velocity and angular velocity of USV were set as *v*(*t*) and *ω*(*t*), respectively. Considering that the interval between two adjacent samplings was very short, the motion in this interval was regarded as a uniform linear motion. According to the projection of this trajectory on the world coordinate system, the position and pose increment of two adjacent moments was expressed as:

(3) $$\{\begin{array}{c} \Delta x=v_{t}\Delta \mathrm{tcos}\left(\theta_{t}\right)\\ \Delta y=v_{t}\Delta \mathrm{tsin}\left(\theta_{t}\right)\\ \Delta \theta =\omega \Delta t \end{array}$$

Then, the position and pose of the USV at time t + 1 was expressed as:

(4) $$X_{b}^{\left(t+1\right)}=\left[\begin{array}{c} x_{b}^{\left(t+1\right)}\\ y_{b}^{\left(t+1\right)}\\ \theta_{b}^{\left(t+1\right)} \end{array}\right]=\left[\begin{array}{c} x_{b}^{t}\\ y_{b}^{t}\\ \theta_{b}^{t} \end{array}\right]+\left[\begin{array}{cc} \Delta t\cos\left(\theta_{b}^{t}\right) & 0\\ \Delta t\sin\left(\theta_{b}^{t}\right) & 0\\ 0 & \Delta t \end{array}\right]\left[\begin{array}{c} v_{t}\\ \omega_{t} \end{array}\right]$$

As shown in Equation (4), the trajectory from time *t* to time *t* + 1 depended on the set of velocities (*v,* *ω*) of the USV. However, the value of this set was not directly given. The velocity *v*(*t*) depended on the initial translation velocity *v*(*t_0_*) and the acceleration in the time interval [*t_0_*, *t*], and the angular velocity *ω*(*t*) was determined by the initial angular velocity and rotation angular acceleration.

b. Kinematic Model Under the Influence of Wave

As mentioned above, the kinematic model of the USV in still water was established. To improve the model, the influence of waves was also introduced into this model. When the USV moved in a sea area with waves, it performed six degrees of freedom (6DOF) under the influence of the waves, which included surging, heaving, swaying, yawing, pitching, and rolling. The motion of heaving and pitching was removed from this model because of only considering the in-plane motion of the USV, but swaying, surging, and yawing were included into the kinematic model due to their essential impact on the navigation and trajectory of the USV [25].

Given the rolling motion mainly exerted impact on the navigation safety of the USV, it will be discussed in the evaluation function later. The wave load could be accurately analyzed using the P-M spectrum, but the tremendous calculation could not meet the real-time requirements of local path planning. Therefore, in this study, Daidola’s conclusion [26] was used to establish the wave load model as follows.

(5) $$\begin{array}{c} \begin{array}{c} \tau_{E}^{wa}=\frac{1}{2}\rho gL\zeta_{D}^{2}\left[\begin{array}{c} \cos\chi C_{XD}\left(\lambda \right)\\ \sin\chi C_{YD}\left(\lambda \right)\\ L\sin\chi C_{ND}\left(\lambda \right) \end{array}\right]\\ C_{XD}=0.05-0.2\frac{\lambda }{L}+0.75{\left(\frac{\lambda }{L}\right)}^{2}-0.51{\left(\frac{\lambda }{L}\right)}^{3}\\ C_{YD}=0.46+6.83\frac{\lambda }{L}-15.65{\left(\frac{\lambda }{L}\right)}^{2}+8.44{\left(\frac{\lambda }{L}\right)}^{3} \end{array}\\ \begin{array}{c} C_{ND}=-0.11+0.68\frac{\lambda }{L}-0.79{\left(\frac{\lambda }{L}\right)}^{2}+0.21{\left(\frac{\lambda }{L}\right)}^{3}\\ \zeta_{D}=h/2\\ \lambda =2\pi /k \end{array}\\ k=\omega^{2}/g \end{array}$$

where *h*, *L*, *λ*, *k*, *ω*, and *g* represent the wave height, length of the USV, wavelength, wave number, wave circular frequency, and gravitational acceleration, respectively.

Combining the mass matrix *M* of the USV with the calculated force and moment ($\tau_{E}^{wa}$) that wave acts on the USV, the acceleration matrix A can be easily calculated with the following formula:

(6) $$A=\tau_{E}^{wa}/M$$

Within a short time interval, *Δt*, when only the wave load was applied, the kinematic model of the USV could be shown in the following formula:

(7) $$X_{w}^{\left(t+1\right)}=\left[\begin{array}{cc} \Delta t\cos\left(\chi_{b}^{t}\right) & 0\\ \Delta t\sin\left(\chi_{b}^{t}\right) & 0\\ 0 & \Delta t \end{array}\right]\left[\begin{array}{c} v_{t}\\ \omega_{t} \end{array}\right]+\frac{1}{2}A\Delta t^{2}$$

c. Kinematic Model Under the Influence of Ocean Currents

Ocean current is a large-scale flow of seawater, one of the fundamental physical phenomena in the marine environment, and is caused by diverse factors. Meteorological institutions generally share their data worldwide. Overall, ocean currents are multidirectional and irregular in space and time. In this study, we assumed that the ocean current remained unchanged during a short period and thereby simplified the impact on USV path planning as a constant disturbance. Given that an ocean current is essentially the movement of water in the ocean, it exerts an impact on an USV by superimposing its own velocity on the velocity of the USV. Therefore, in this study, the kinematic model of the USV under the influence of an ocean current is expressed as follows:

(8) $$X_{c}^{\left(t+1\right)}=\left[\begin{array}{cc} \Delta t\cos\left(\theta_{c}^{t}-\theta_{b}^{t}\right) & 0\\ \Delta t\sin\left(\theta_{c}^{t}-\theta_{b}^{t}\right) & 0\\ 0 & \Delta t \end{array}\right]\left[\begin{array}{c} v_{c}^{t}\\ \omega_{c}^{t} \end{array}\right]$$

where $v_{c}^{t},\;\omega_{c}^{t},\theta_{c}^{t}$ represent the current’s velocity, angular velocity, and direction angle, respectively.

Finally, the following complete kinematic model was formed based on Equations (4), (7) and (8).

(9) $$X^{\left(t+1\right)}=X_{b}^{\left(t+1\right)}+X_{w}^{\left(t+1\right)}+X_{c}^{\left(t+1\right)}$$

#### 2.2.2. Evaluation Function

In the determined search space *V_r_*, multiple velocity combinations and their corresponding trajectories were obtained by discrete sampling. However, the optimal path should meet the requirements of shortening distance, effectively avoiding obstacles, and quickly reaching the target position. Therefore, it was necessary to construct an evaluation function to determine the best path from those obtained groups of motion trajectories. The evaluation function of the classical DWA consists of three weighted terms [27,28,29]:

(10) P(v,ω)=σ(α⋅heading(v,ω)+β⋅dist(v,ω)+γ⋅vel(v,ω))

As discussed above, rolling was not considered in the process of analyzing the influence of waves on the USV in the kinematic model. In this model, rolling did not directly affect the heading and trajectory of the USV but significantly affected its controllability and stability. Therefore, the above simple evaluation function did not meet the requirements. To improve the safety of navigation, an additional item, sailing (*v*, *ω*), was added to the evaluation function, and then a minimum score was given for the dangerous situation where the heading and wave directions were perpendicular and may have caused capsizing. In addition, a score was given for hazardous situations where the heading and wave directions were perpendicular and may have led to capsizing.

(11) $$sailing\left(v,\omega \right)=\left(1+\left|\cos\left(\theta_{b}-\theta_{w}\right)\right|\right)v_{w}$$

Then, the final evaluation function was achieved, as follows, by adding Equation (11) to Equation (10):

(12) P(v,ω)=σ(α⋅heading(v,ω)+β⋅dist(v,ω)+γ⋅vel(v,ω)+δ⋅sailing(v,ω))

## 3. Simulation Results and Discussion

All simulations in this study were carried out using the MATLAB 2017A version on the PC with a Windows 10 system. Intel i5 3.4 GHz quad-core CPU and 8 GB RAM were built into the PC. The USV in this study featured a rectangular rigid body, and its basic parameters are given in Table 1 below.

**Figure 3:**
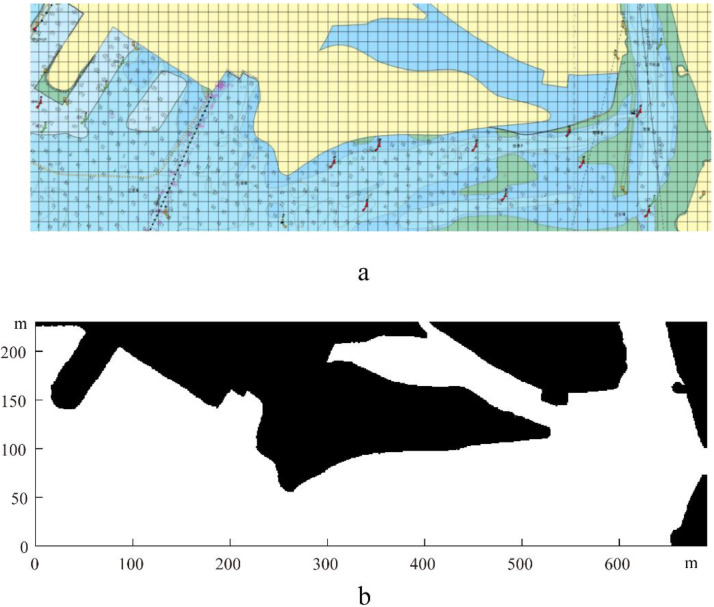
A map (**a**) and its corresponding grid map (**b**).

To verify the effectiveness of the method discussed above, a port in the Bohai region of China was selected as the research site. Its map is shown in Figure 3a with a size of 690 m × 230 m, and its grid map is shown in Figure 3b.

**Figure 4:**
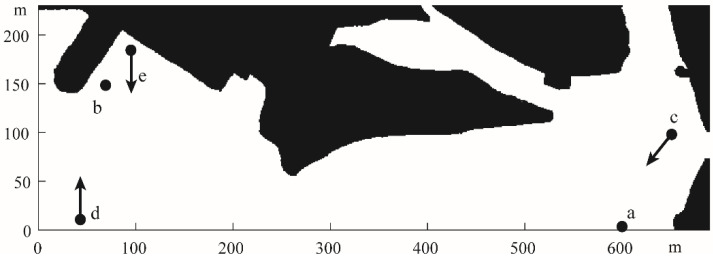
Obstacle settings. Where a and b are the navigation’s starting point and endpoint, respectively, and c, d and e are the dynamic obstacles.

Figure 4 illustrates the detailed setting of the simulation experiment, where a and b are the navigation’s starting point and endpoint, respectively, and c, d and e are the dynamic obstacles in this simulation. At *t* = 0, c, d, and e started moving in a straight line in the direction of 220°, 90° and −90° at a speed of 7.8 m/s, 0.6 m/s and 0.3 m/s, respectively. In the end, we evaluated the effectiveness of this method in terms of the generation of the final path, the path length, and the navigation time.

### 3.1. Simulation Results under the Action of Waves in Different Directions

According to the statistical analysis results of the marine environment of the Bohai Sea in the literature [30], this study set the environmental parameters as a wave height of 1 m, a wave period of 3 s, and a current velocity of 1.5 knots. Then, the simulation experiments were carried out in three situations of no current and wave (still water), upward loads, and downward loads (the included angles between the direction of environmental loads and the connecting line from the USV’s starting point to the ending point were acute angle and obtuse angles, respectively).

**Figure 5:**
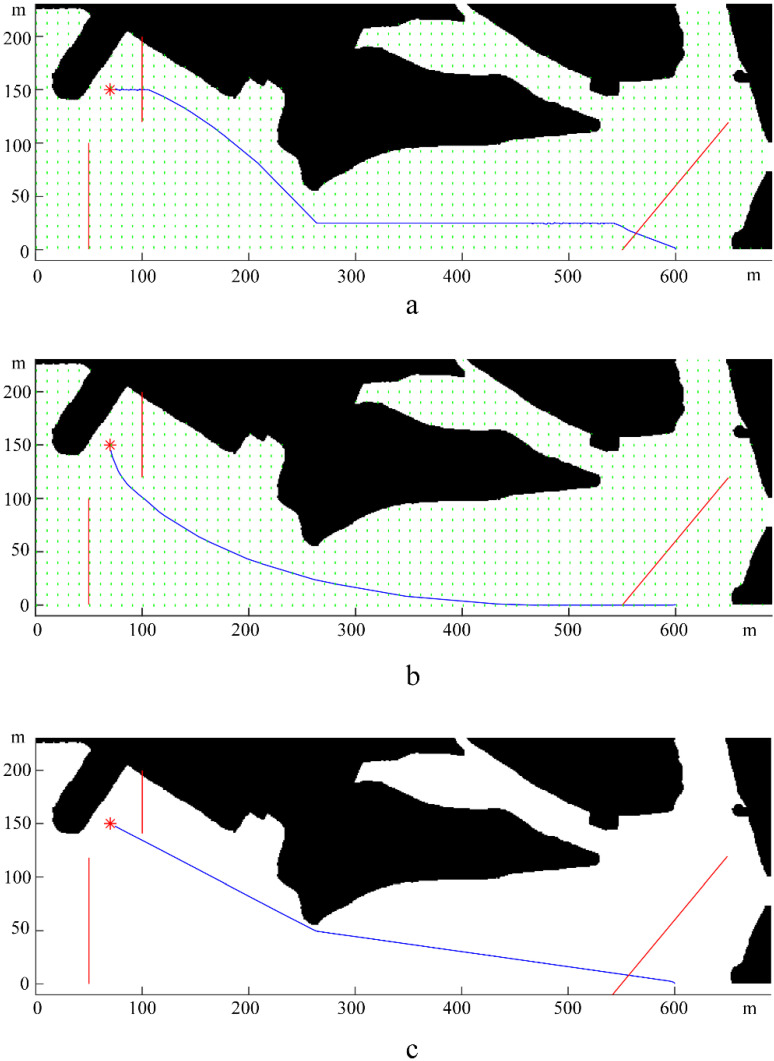
Final path under upward loads (**a**), downward loads (**b**), and in still water (**c**). Where “*” represents for the final destination, the red lines represent for the paths of dynamic obstacles, and the blue line represents for the simulated navigation path in each subfigure.

Figure 5 shows the calculated path of the USV under the mentioned three environmental conditions. The paths (Figure 5a,b) under wave loads were entirely different from the one under still water (Figure 5c), which proved that the environmental loads indeed had a significant impact on path planning. In addition, since the different directions of environmental loads led to different resistance and energy consumption during USV navigation, the path planning results under upward and downward environmental loads (corresponding to Figure 5a and Figure 5b, respectively) were also significantly different from each other. Moreover, the path planning algorithm reduced energy consumption by adjusting the heading angle reasonably, which also resulted in the different final calculated paths.

**Figure 6:**
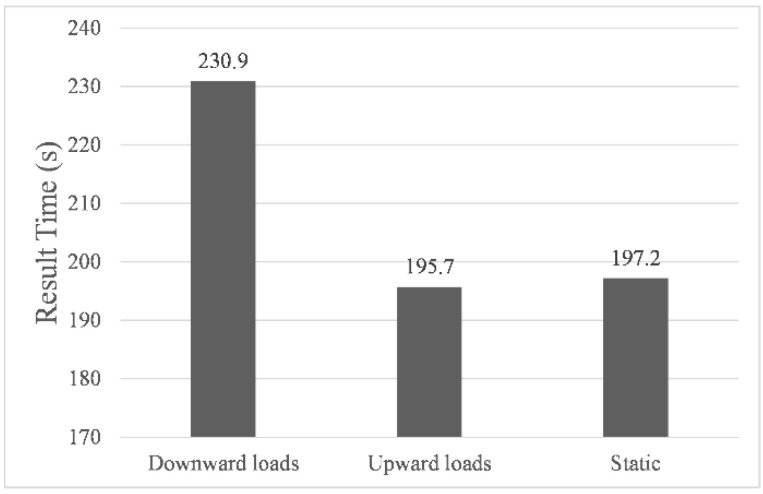
Navigation time.

**Figure 7:**
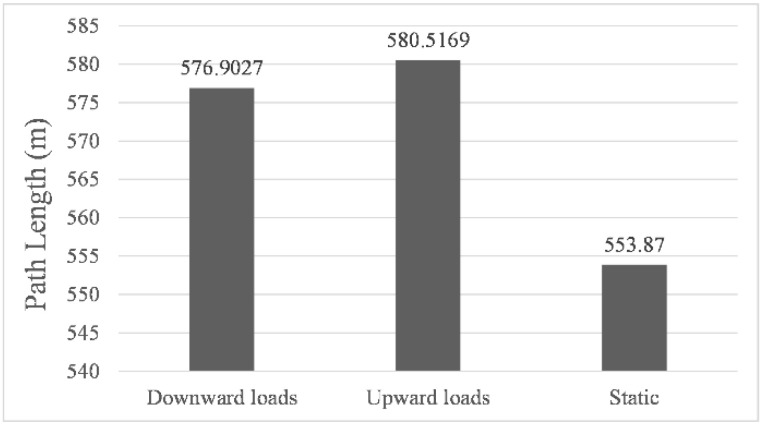
Path length for simulated navigations.

**Figure 8:**
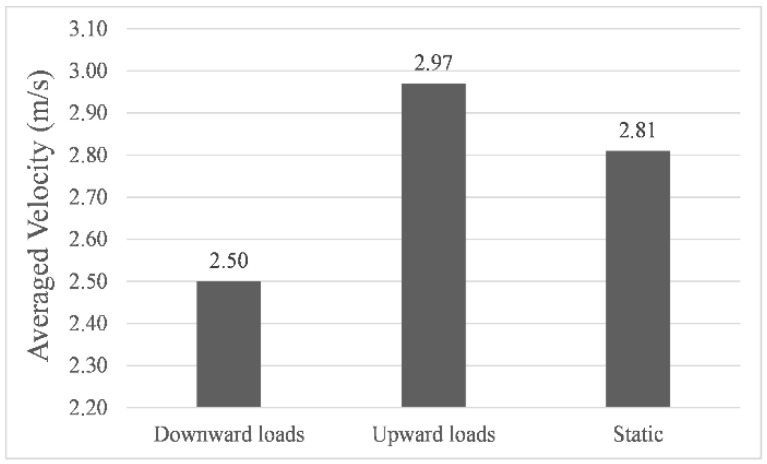
Averaged velocity of simulated navigations.

Figure 6, Figure 7 and Figure 8 showed the results of navigation time, path length, and average speed calculated under different environmental load conditions, respectively. Taking the results in still water as benchmarks, in upward load, the navigation time was shortened to 99.2%, the path length increased to 104.8%, and the average speed increased to 105.7%, while in downward load, the navigation time was prolonged to 117.1%, the path length increased to 104.2%, and the average speed was reduced to 88.9%. The above results indicate that the direction of the environmental load had a significant impact on the calculated navigation time and the shape of the final path, and the introduction of environmental load indeed enabled us to calculate the planning path with different parameters. It is worth noting that when the direction of the environmental load changed by 180°, the difference in path length calculated under upward and downward load conditions was only 3.6142 m, which proved that its correlation with the direction of the environmental load was very weak.

### 3.2. Simulation Results under the Action of Waves and Currents with Different Intensities in the Same Direction

**Figure 9:**
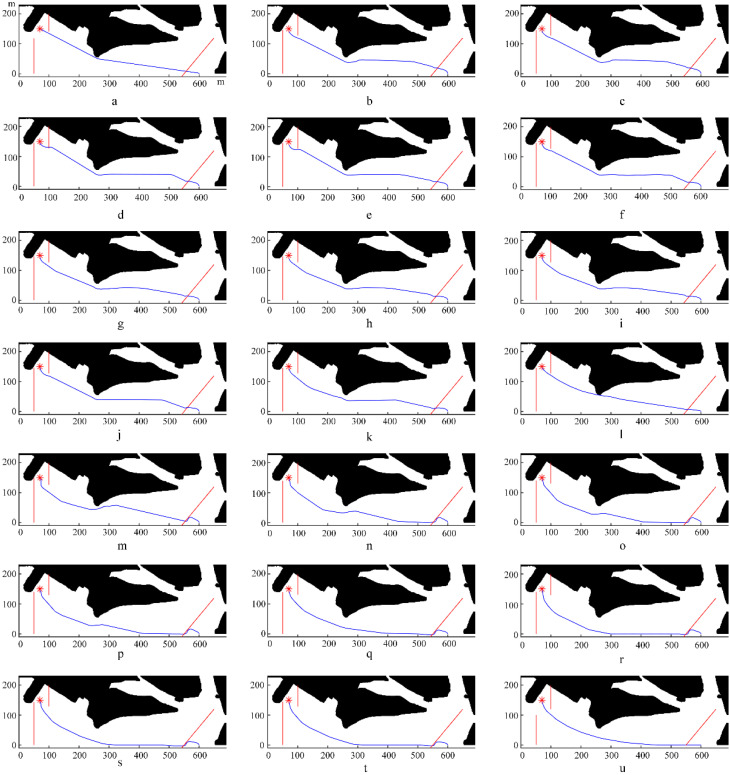
Final simulated paths under environmental factors with different intensities ((**a**–**u**), the intensity coefficient gradually increases from 0 to 1 in 0.05 intervals). In each subfigure, “*” represents for the final destination, the red lines represent for the paths of dynamic obstacles, and the blue line represents for the simulated navigation path.

In the above, waves and currents with fixed intensity and different directions were used to test the effectiveness of our algorithm. This part will investigate the effect of environmental load intensity on the calculated USV path planning using the waves and currents with fixed direction and different intensities. In terms of the experimental parameters, the direction of waves and currents was set as −90°, and the environmental factor intensity set previously (wave height of 1 m; current velocity of 1.5 knots) was regarded as 1X. Then, starting from 0X, the environmental factor intensity gradually increased with intervals of 0.05X to check its impact on the planned paths. In Figure 9, the final simulated paths were arranged in ascending order according to the intensity of the environmental factors. The intensity multiples corresponding to Figure 9a,u were 0X and 1X, respectively. Table 2 summarizes the parameter results obtained from the simulation experiments under environmental factors with different intensities.

**Figure 10:**
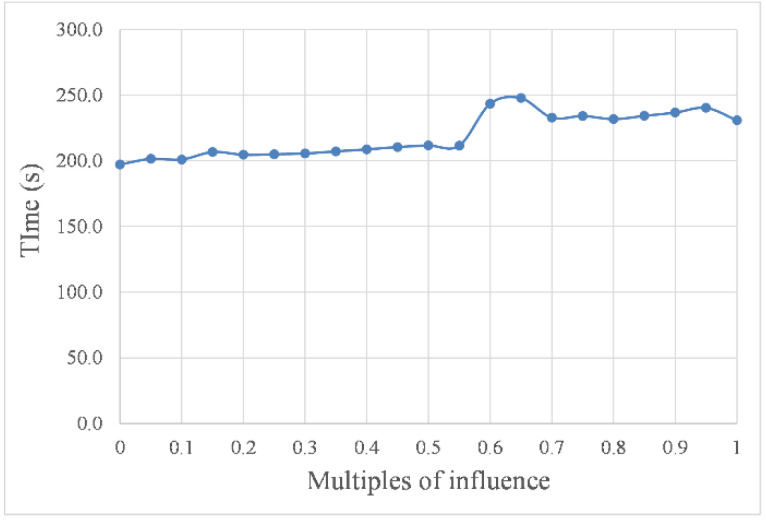
Navigation time with different intensities.

**Figure 11:**
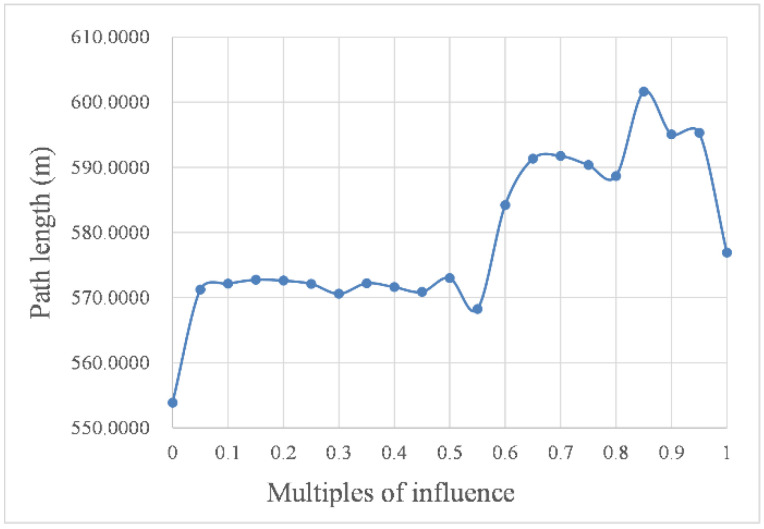
Path length affected by different intensities.

To visualize the influence of environmental factor intensity changes on the path planning parameters, we transformed the parameters in Table 2 into the curves in Figure 10 and Figure 11 to represent the navigation time and path length under different intensities of environmental factors, respectively.

As shown in Figure 10 and Figure 11, with increasing intensity, the impact of the marine environment factors on USV path planning resulted in a step-like curve. In other words, when the intensity was within the threshold, the value of the influence remained stable, but when the intensity was out of this threshold, the value of the influence changed significantly. For example, the curves in the abscissa range of 0.05 to 0.5 were almost horizontal in Figure 10 and Figure 11, and the corresponding planned path shapes, as shown in Figure 9b–k, were very similar. However, these curves had a sharp change at the abscissa of 0.5. In addition, compared with the curves in the abscissa range of 0.05 to 0.5, the curves in the abscissa range of 0.55 to 1.00 had a more significant change. Taking the path length in still water as the benchmark, the change rate of the path length caused by environmental factor load was as high as 8.6%.

Remarkably, the curve in the abscissa range of 0.8 to 0.9 in Figure 11 had a significant rise, and the corresponding simulated paths in this range are shown in Figure 9q–s. As shown in the simulated paths of the three simulation experiments, the influence of moving obstacle c on path planning causes a significant increase, with the result that the USV cannot quickly leave the area where it may collide with c. Therefore, the algorithm prefers to reserve a safe enough distance to ensure that the USV will not collide with the obstacle.

After comprehensively analyzing the above data groups, we summarized the impacts of environmental factors on USV local path planning as follows. First, the direction of marine environmental factors exerted little impact on the final path length, but it significantly changed the speed of the USV, thus affecting the navigation time. Second, the impact of the intensity of marine environmental factors on USV path planning performed a step-like change at the threshold determined by the navigation performance of the USV.

This method systematically considers the impact of waves and currents on USVs, which ensures that USVs can adjust the planned path in time and avoid obstacles when navigating in the real marine environment.

### 3.3. Computing Time

**Figure 12:**
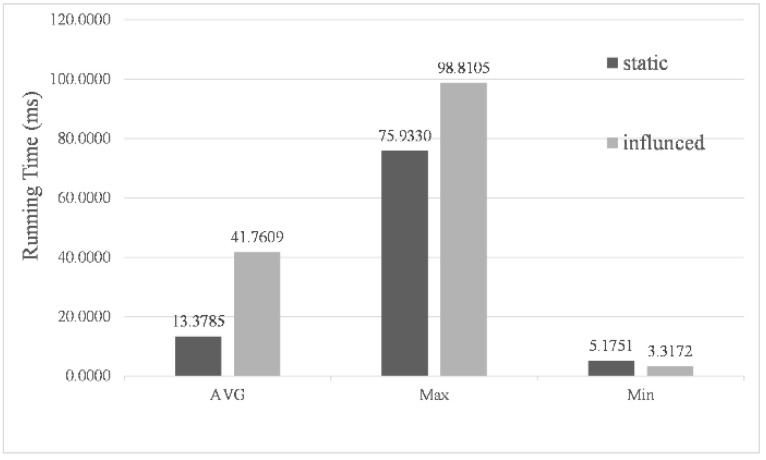
Program calculates time per statistical cycle analysis.

In the simulation experiments, the actual navigating time corresponding to each cycle of the algorithm was 0.1 s. Considering the practical significance of the algorithm, the calculation time of each cycle must be less than 0.1 s to ensure the real-time update for the navigation path of the USV. Figure 12 shows the statistical results of the calculation time for 50,113 cycles under the presence (influenced) and absence (static) of environmental factor impact. Due to considering environmental impact, the kinematic model and the evaluation function were more complex, thus increasing the calculation time of the algorithm. Nevertheless, the average calculation time of each cycle was only 0.0418 s, with the longest time no more than 0.1 s, which fully proves the real-time performance of the algorithm.

## 4. Conclusions

In this study, considering the significant impact of wave and current loads on USVs, we redesigned the kinematic model and then proposed an improved DWA algorithm for the local path planning of an USV. Finally, using the simulation method, we comprehensively evaluated the practicability and effectiveness of this method in terms of navigation time, path length, and program operation time.

The simulation results showed that the navigation time of the USV was affected by the direction and intensity of the environmental load, but the path length of the USV was only slightly affected by the direction of environmental load and greatly affected by its intensity. Specifically, when the direction of the environmental load changed by 180°, compared with the situation in still water, the path length only changed 0.65%, but the navigation time changed as significantly as 17.8%. The load direction had an important impact on the navigation time and the final path shape, since it affected the heading angle selected by the algorithm. Given that the change in the intensity of environmental factors significantly impacted both the path length and navigation time of the USV, further changes in both at certain intensity thresholds may depend on the navigation performance of the USV. Moreover, the average and maximum times of each calculation cycle were less than the actual navigation time.

Last but not the least, this method can adjust the planning path of USVs in a timely manner according to ocean waves and currents, thereby offering good practicability and real-time performance.

## Figures and Tables

**Table 1:** The basic parameters of the USV studied in this work.

Total Mass	Length	Width	Maximum Speed	Maximum Angular Speed	Acceleration Range	Angular Acceleration Range
100 kg	2.88 m	1.30 m	3 m/s	360°/s	−1~2 m/s^2^	−360~360°/s^2^

**Table 2:** Results obtained from simulation experiments under environmental factors with different intensities.

Multiples of Intensity	Simulated Navigation Time (s)	Simulated Path Length (m)
0.00	197.2	553.8700
0.05	201.5	571.2285
0.10	201.0	572.1262
0.15	206.7	573.1262
0.20	204.6	573.7434
0.25	205.0	574.7434
0.30	205.6	575.7434
0.35	207.2	576.7434
0.40	208.7	572.7434
0.45	210.5	572.7434
0.50	211.7	572.7434
0.55	211.6	564.2380
0.60	243.5	584.1969
0.65	247.8	591.3040
0.70	232.9	591.7422
0.75	234.2	590.3542
0.80	231.8	588.6524
0.85	234.3	601.6053
0.90	236.8	595.0392
0.95	240.4	595.3041
1.00	230.9	576.9027

## Data Availability

All the data supporting the reported results has been included in this paper.
